# The Role of Stress Regulation on Neural Plasticity in Pain Chronification

**DOI:** 10.1155/2016/6402942

**Published:** 2016-12-08

**Authors:** Xiaoyun Li, Li Hu

**Affiliations:** ^1^Key Laboratory of Cognition and Personality, Ministry of Education and Faculty of Psychology, Southwest University, Chongqing, China; ^2^CAS Key Laboratory of Mental Health, Institute of Psychology, Beijing, China

## Abstract

Pain, especially chronic pain, is one of the most common clinical symptoms and has been considered as a worldwide healthcare problem. The transition from acute to chronic pain is accompanied by a chain of alterations in physiology, pathology, and psychology. Increasing clinical studies and complementary animal models have elucidated effects of stress regulation on the pain chronification via investigating activations of the hypothalamic-pituitary-adrenal (HPA) axis and changes in some crucial brain regions, including the amygdala, prefrontal cortex, and hippocampus. Although individuals suffer from acute pain benefit from such physiological alterations, chronic pain is commonly associated with maladaptive responses, like the HPA dysfunction and abnormal brain plasticity. However, the causal relationship among pain chronification, stress regulation, and brain alterations is rarely discussed. To call for more attention on this issue, we review recent findings obtained from clinical populations and animal models, propose an integrated stress model of pain chronification based on the existing models in perspectives of environmental influences and genetic predispositions, and discuss the significance of investigating the role of stress regulation on brain alteration in pain chronification for various clinical applications.

## 1. Introduction

Chronic pain is a main source of worldwide disability, causing physical and psychological discomforts and rising huge medical expenses [1]. Understanding mechanisms of the development of chronic pain is crucial in monitoring and preventing the progress of pain chronification. In recent decades, increasing clinical studies and complementary animal models contributed to important advances in understanding the transition from acute to chronic pain. Notably, Melzack [2] proposed that stress played an important role in such pain chronification, and accumulating evidence demonstrated that stress regulation (as indexed by the function of hypothalamic-pituitary-adrenal [HPA] axis) consistently engaged in the development of chronic pain [3–5]. In line with these findings, several brain regions, subserving as key candidates for stress regulation [6–8], have been reported to be involved in the transition from acute to chronic pain, including the amygdala, prefrontal cortex (PFC), and hippocampus [9–12]. Therefore, some previous studies hypothesized that these brain regions, especially within the emotional corticolimbic system, acted as the bridge of pain modulation and stress regulation. In this paper, we briefly walk through concepts of pain and stress, review effects of the HPA function on acute and chronic pain, and discuss alterations of stress-associated brain regions in acute and chronic pain. In the following, we discuss two existing stress models of chronic pain in perspectives of environmental influences and genetic predispositions, respectively, and propose an integrated stress model of pain chronification based on previous findings.

## 2. Acute Pain and Chronic Pain

Pain is a conscious sensation, processing multidimensional information involving sensory, affective, and cognitive components [2]. Acute pain, serving as a warning of injury or illness, functions to protect the organs from a present or potential damage. If acute pain continues for a long time (e.g., longer than 3 months), it can develop into chronic pain, even after the initial injury or illness has been healed [13]. In addition, the chronic pain itself is usually associated with hyperalgesia and/or allodynia [14] and psychological distress, such as anxiety and depression [15].

## 3. Stress and the HPA Axis

Stress is a biological reaction [2] that triggers a rapid response by activating the sympathetic nervous system and a relative slower response via evoking the HPA axis [8]. The faster pathway releases catecholamines, such as adrenaline and noradrenaline, priming the body into a classic “fight or flight” mode with enhanced activation of the sympathetic nervous system (e.g., increased heart rate and blood pressure, sweat gland activation). When the slower stress response pathway of the HPA axis is activated, corticotropin-releasing factor (CRF) travels from the paraventricular nucleus (PVN) of the hypothalamus to the pituitary, leading to the release of adrenocorticotropic hormone (ACTH). In turn, ACTH stimulates the adrenal gland to secrete glucocorticoids (cortisol in humans, corticosterone in rodents) that are essential for stress response. This type of hormone can naturally go through the blood-brain barrier and access multiple brain regions, like the amygdala, PFC, and hippocampus, binding with two intracellular receptors, the glucocorticoid receptor and the mineralocorticoid receptor [16]. As such, glucocorticoids can influence neuronal excitability and synaptic and neuronal plasticity [17, 18].

## 4. The Effect of Stress Regulation on Pain

Studies associated with stress responses have demonstrated that altered activations of the HPA axis are in response to experimental pain [19, 20] and various chronic pain disorders [3, 5]. Conceptually, pain-related activation of the HPA axis has been embedded in an allostatic load model of disease [4, 21]. This model assumes that, in response to acute stress, various physiological activities could be activated to help organism adapt to environmental changes. It has also been proposed that acute pain induced cortisol elevation may reduce pain unpleasantness [22] and increase pain tolerance [23], which provides solid evidence for the transient stress-induced analgesia [24]. However, when stress, induced by physical injury and/or pain-related psychological factors, is prolonged, uncertain, and uncontrollable, the response of such stress becomes maladaptive, entering into a vicious cycle, in which acute pain has evolved into a chronic state with abnormal alterations of brain structures and functions [4, 21]. Meanwhile, cortisol fails to act its protective functions under chronic pain conditions, thereby exaggerating pain severity. In fact, emerging evidence suggests that chronic pain is associated with the dysfunction of cortisol secretion, although the nature of their relationship is not fully elucidated. A majority of previous studies reported reduced cortisol secretions or lower basal levels of cortisol in various chronic pain disorders, such as fibromyalgia [25, 26], chronic whiplash-associated disorder [27], chronic neck pain [28], chronic low back pain [29], chronic fatigue syndrome [30], and chronic pelvic pain [31]. Since the HPA axis is a self-regulating negative feedback system [7], such hypocortisolism is indicative of attenuated activity or impaired feedback sensitivity of the HPA axis. However, a few studies found elevated cortisol levels [32–35], abnormal cortisol diurnal variations [36], and increased feedback sensitivity of the HPA axis [37] in certain chronic pain conditions, like fibromyalgia and chronic back pain. In contrast, several studies reported that the profiles of cortisol secretion in patients suffering with fibromyalgia [38, 39], chronic back pain [40, 41], or chronic temporomandibular disorders [42] did not differ from those in healthy controls. One possible explanation to these conflicting findings is that the HPA axis may be in the state of hyperactivity at the early stage of pain chronification, while, after long-term overt activity, the stress system reaches an exhausted state, thereby turning into the HPA axis hypoactivity [26]. Albeit such explanation requires further verification, it is reasonable to suggest that the HPA axis is involved in the development of chronic pain.

## 5. Neural Plasticity in Acute and Chronic Pain

The corticolimbic system, including amygdala, PFC, and hippocampus, is a powerful neural network that has been suggested to contribute to the transition from acute to chronic pain [12]. Further, both acute and chronic pain profoundly influence this system, which is also known to relate to stress regulation [8], via structural and functional alterations in the related brain regions. Compelling evidence supporting this conclusion was well documented by various human neuroimaging studies and animal models.

### 5.1. Brain Responses to Acute Pain

Most human neuroimaging studies on acute pain have revealed consistent activations in the insula and dorsal anterior cingulate cortex [43–45]. Even less consistency, activations in the amygdala, PFC, and hippocampus have been frequently reported in neuroimaging studies of acute pain [46–51]. Specifically, noxious stimulation applied on pain-free individuals evoked stronger Blood-Oxygen-Level-Dependent signals in the PFC [46, 51–53] and hippocampus [46, 48, 54, 55]. Besides, activations of amygdala and hippocampus were observed to be related to pain expectancy [53, 55, 56]. Furthermore, augmented activation of amygdala was induced by pain in depressed individuals [57] and in healthy cohort with greater pain unpleasantness followed by an induction of depressive mood [52], suggesting the role of amygdala in the integration of pain and emotional information. In addition, the HPA axis influences the activations of the abovementioned brain regions under acute pain conditions. For instance, previous studies demonstrated that elevated cortisol levels were (1) associated with reduced pain unpleasantness and decreased pain-related brain activation during constant noxious stimulation [22], (2) linked with lower pain threshold and stronger PFC activity in response to inflammation-induced pain [58], and (3) related to enhanced hippocampal activation during step-up noxious stimulation (an increasing pattern of noxious stimulation) [59]. These studies suggested that acute pain, acting in a similar way with acute stress, may evoke cortisol levels to boost the survival of the organism by inhibiting and/or facilitating activities of related brain networks. Although no path analysis in these studies was performed to verify the interrelationship among acute pain, stress regulation, and functional changes of the corticolimbic system, it is highly likely that acute pain may evoke an adaptive response to protect the organism via the coinfluence of cortisol elevation and brain responses in the amygdala, PFC, and hippocampus.

In fact, evidence from animal models suggests that this may be the same case. Consistent observations of acute pain-related functional changes in the corticolimbic system have been reported in animal studies. For instance, noxious stimulation induced neuronal excitability in the amygdala [60–62] and PFC [63] and neuronal inhibition in the CA1 hippocampus [64, 65]. In line with the electrophysiological findings, immunohistochemical and fMRI studies also showed similar activation patterns in these brain regions by delivering noxious stimulation on rats [63, 66–69]. These findings confirmed the role of acute pain on brain plasticity. Additionally, it is important to find out the role of stress regulation in the changes of brain responses under acute pain conditions, and evidence from an immunocytochemical study might shed light on this issue [70]. In this study, rats with less pain sensitivity showed less freezing responses, stronger vocalization, and increased PFC activation and plasma corticosterone levels, in response to conditioned aversive stimuli. The authors also found an increased Fos expression (an indicator of neuronal activity in rats) in the hypothalamus and dentate gyrus of the hippocampus in the same group of rats. In contrast, rats with high pain sensitivity responded more passively to aversive events (e.g., more freezing behavior and weaker vocalization), along with increased activation in the amygdala and CA1 hippocampus, but did not show significant changes in corticosterone levels [70]. These findings suggested that enhanced activity of stress axis in rats with low pain sensitivity was due to the role of the PFC and hippocampus in regulation of glucocorticoid release, which was crucial for survival, while such regulation was ineffective in rats with high pain sensitivity [70].

In summary, human and animal studies of acute pain have provided evidence in support of the viewpoint that acute pain, similar to acute stress, increased the release of glucocorticoid. Meanwhile, the above discussed brain regions are highly sensitive to acute painful and stressful stimuli and are highly plastic via regulation of the glucocorticoid negative feedback in the PFC and hippocampus. Therefore, it is conceivable that the whole process can be considered as the reaction in an adaptive and protective response system to aversive stimuli, leading to a better adaptation for survival.

### 5.2. Brain Alterations in Chronic Pain

Human neuroimaging studies have emphasized the association between chronic pain and abnormal changes in gray matter volume and thickness of the brain in various chronic pain patients [71–73]. Remarkably, a large number of studies have repeatedly showed reduced volumes of the amygdala [12, 74–78], medial prefrontal cortex (mPFC) [71, 74, 79–81], and hippocampus [32, 71, 81–83] in a variety of chronic pain populations. In accordance with the morphological alterations, functional changes of the amygdala, mPFC, and hippocampus were also observed under chronic pain conditions [47, 84, 85]. A meta-analysis study reported that peak activation was found in the basolateral amygdala in clinical populations, suggesting the enhanced cognitive-affective processing among patients suffering from chronic pain [85]. In chronic pain patients, clinical pain intensity appeared to be associated with greater activations in the mPFC [86, 87] and hippocampus [32]. Additionally, under the circumstance of clinical pain fluctuation, greater functional connectivity of mPFC with the limbic system, including the amygdala and hippocampus, was indicative of pain chronification [9, 10, 12, 85, 88]. Although stress regulation and pain chronification are suggested to share a similar mechanism in modulating brain structures and functions [2], how stress regulation plays a role in brain alterations in patients with chronic pain remains unclear. Recently, a clinical study explored the influence of maladaptive stress response on pain state of patients, such as elevated cortisol levels that were associated with enhanced clinical pain intensity, smaller hippocampal volume, and stronger hippocampal activations [32], which provided strong evidence to demonstrate the important role of the maladaptive stress response on the transition from acute to chronic pain [4]. Interestingly, a recent study of chronic myofascial pain reported that gray matter atrophy in the PFC and hippocampus was independent of the cortisol levels. They also reported that only the gray matter density of PFC was negatively correlated with pain thresholds in patients, suggesting the presence of pain disinhibition [89]. Despite these findings are contradictive, it can be imaginable that dysfunction of the HPA axis and abnormal brain alterations may be caused by maladaptive responses to chronic pain.

In support of the above viewpoints, evidence from animal studies has unraveled the role of chronic pain in altering the structure and function of the corticolimbic system. The long-lasting sympathic pain increased neuronal excitability and dendritic branching of amygdala in rodents [90, 91], resulting in an enlarged amygdala volume [92]. Spared nerve injury sympathic pain induced alterations of dendritic length, spine density, and neuronal activity in the mPFC [93, 94], and long-term neuropathic pain reduced prefrontal volumes in rodents [95]. Moreover, hippocampal neurogenesis appeared to be suppressed in a rat model of neuropathic pain, subserving a possible mechanism of pain chronification [83, 96]. Additionally, since the mPFC receives inputs from both amygdala and hippocampus [97], pain-related amygdala hyperactivity inhibited the mPFC activation and impaired functions related to decision-making in an arthritis pain model [98, 99], whereas reduced hippocampus-prefrontal connectivity was associated with impaired spatial memory performance in rats with neuropathic pain [100]. Yet the knowledge of influence of stress regulation on brain alterations in chronic pain is very limited. An animal study of chronic neuropathic pain reported that enhanced nociceptive sensitivity during chronic pain was associated with increased activation in the amygdala and decreased activation in the hippocampus but failed to influence activation of the HPA axis [101]. Interestingly, such dissociation between pain sensitivity and stress response in rats implicated that the HPA axis dysfunction in chronic pain patients might not originate from pain itself, but rather from other factors associated with repeated painful stimuli (e.g., experimental cues and an inability to escape from pain).

In summary, human neuroimaging studies and animal models of chronic pain have suggested that chronic pain could induce a chronic stress-like alteration in the HPA axis and the corticolimbic system, with dysregulation of the HPA axis, and dysfunction and reorganization of the corticolimbic system. It is reasonable to conclude that such maladaptive responses are likely to contribute to the development of chronic pain.

## 6. Stress Models of Pain Chronification

Given that activation of the HPA axis influences neural alterations, both structurally and functionally, the role of stress regulation becomes critical in the understanding of brain mechanisms in the evolution of acute pain into chronic pain.

From the perspective of stress regulation, human and animal studies suggest that chronic stress has an enduring and destructive effect on the brain via activation of the HPA axis, particularly the glucocorticoid secretion [102]. To explain this phenomenon, two different hypotheses have been proposed: the neurotoxicity hypothesis and the vulnerability hypothesis. The neurotoxicity hypothesis suggests that prolonged release of glucocorticoid impairs the neuronal capacity to resist toxic invasion or normal attrition, leading to the reduction of hippocampal volume in populations with chronic stress, including posttraumatic stress disorder (PTSD) and depression [103]. In contrast, the vulnerability hypothesis points out that small hippocampal volume is a predetermine risk factor for chronic stress that is shaped by genetic predispositions and/or early life stress [104], as evidenced in the studies of stress [105], anxiety [106], and PTSD [104].

Accordingly, two stress models of chronic pain have also been proposed, from environmental influence and genetic predisposition perspectives, respectively: a “neurotoxic model” and a “vulnerability model.” The “neurotoxic model” suggests that persistent pain may lead to the maladaptive stress response, such as dysfunction of the HPA axis, affecting the alterations in brain structure and function [6, 107]. In line with this model, chronic pain-related changes in the corticolimbic system, including the amygdala, PFC, and hippocampus [47, 71, 73, 108], have been considered as a consequence of allostatic load of chronic disease, in which prolonged pain dysregulates the HPA axis, thereby impairing brain structures and functions [4]. In contrast, the “vulnerability model” proposes that the characteristics of some particular brain structures, such as the volume of the brain regions within the corticolimbic system [109], may contribute to the vulnerability of development from acute to chronic pain, thus affecting the activation of the HPA axis and brain functions [105, 110]. For example, a longitudinal study tracked brain alterations for 3 years in patients with subacute back pain as they gradually evolved to chronic pain states and found that small sizes of amygdala and hippocampus were the preexisting risk factors in the development of chronic back pain [12].

**Figure 1:**
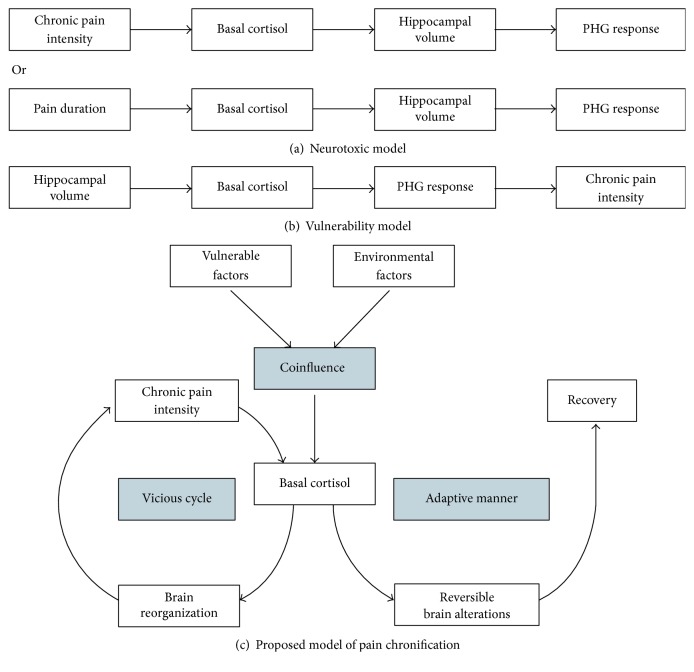
Stress models of chronic pain. (a) The neurotoxic model, from the perspective of environmental influences, conceptualizes that chronic pain intensity or pain duration may lead to the maladaptive stress response, affecting the structure and function of the hippocampal formation and the parahippocampal gyrus (PHG) [32]. (b) The vulnerability model, from the perspective of genetic predispositions, conceptualizes that the small hippocampal volume as a vulnerable factor affects the levels of stress hormones, which in turn lead to enhanced activations of the parahippocampal gyrus and increased persistent pain intensity [32]. (c) The proposed integrated model, from the perspective of the combination of environmental influences and genetic predispositions, conceptualizes that vulnerable factors (e.g., properties of particular brain structures) and environmental factors (e.g., injury) codetermine the levels of basal cortisol, which result in the brain alterations. Thereafter, these physiological responses either return to normal levels with an adaptive manner or initiate a vicious cycle of cortisol dysfunction, brain reorganizations, and persistent pain state.

It is of interest to note that a recent study of chronic back pain using path analysis has suggested that small hippocampal volume has represented a risk factor of vulnerability to persist pain in a maladaptive stress response manner (the “vulnerability model”) [32]. However, their observation cannot disprove the “neurotoxic model” because the development of chronic back pain, similar to other chronic pain diseases, is a dynamic process, and any cross-sectional study cannot track the causal relationship among pain chronification, stress regulation, and brain alterations during the whole process. Indeed, we are not able to rule out the possibility that the influence of stress response in pain chronification is driven by the combination of both environmental and genetic factors. It should be noted that emerging studies in the perspectives of stress regulation or pain chronification support this possibility [102, 111]. We presume that the level of activation of the HPA axis is determined by the combination of vulnerable factors (e.g., structural characteristics of the corticolimbic system) and environmental factors (e.g., pain caused by an injury) and influences the alterations of the structure and function of the corticolimbic system. Subsequently, these physiological responses would lead to either the recovery of health or the persistence of pain state that in turn strengthens brain reorganizations and eventually develops into a chronic pain state (Figure 1). Besides, the proposed stress model of pain chronification should be verified in a longitudinal design by tracking the alterations of all physiological responses, including brain structures and functions, and glucocorticoid levels. We foresee that unraveling the stress effect on brain mechanisms in pain chronification will be of great importance to predict the development of chronic pain [9–12], thus having important clinical significance.

## 7. Conclusion

We have provided a broad range of compelling evidence that pain modulation and stress regulation can be considered as an integrated processing [2], within the framework of corticolimbic system. Human neuroimaging studies and animal models have unfolded a field of vision in the mechanism that drives the development of chronic pain under the HPA axis regulation. In contrast to the fact that activations of the HPA axis and the corticolimbic system show an adaptive manner to the perceived danger in acute pain, chronic pain is commonly associated with dysfunction of the HPA axis and brain reorganizations. Based on the two existing stress models of chronic pain, we propose an integrated stress model of pain chronification, emphasizing the integration of contingent environmental influences and genetic predispositions in the transition from acute to chronic pain. However, the precise mechanism of this integrated model remains to be investigated. Hopefully, future studies can determine physiological responses in relation to activation of the HPA axis and alterations of the corticolimbic system as the specific biomarkers of pain chronification, for monitoring and preventing the transition to chronic pain. The identification of these biomarkers in patients suffering from acute and chronic pain is important, as it can help facilitate the effective pain rehabilitation, reduce pain-related disability, and improve quality of life.
